# Complete Genome Sequence of *Escherichia coli* Strain RS218 (O18:H7:K1), Associated with Neonatal Meningitis

**DOI:** 10.1128/genomeA.00804-15

**Published:** 2015-07-23

**Authors:** Dona Saumya S. Wijetunge, Robab Katani, Vivek Kapur, Subhashinie Kariyawasam

**Affiliations:** a Department of Veterinary and Biomedical Sciences, The Pennsylvania State University, University Park, Pennsylvania, USA; b Center for Molecular Immunology and Infectious Disease, The Pennsylvania State University, University Park, Pennsylvania, USA

## Abstract

*Escherichia coli* RS218 is the prototypic strain of neonatal meningitis-causing *E. coli* (NMEC) and has been used in many studies related to NMEC pathogenesis. In the present study, the genome of *E. coli* RS218 was sequenced together with its plasmid, pRS218. Here, we report the fully closed genome sequence of *E. coli* RS218.

## GENOME ANNOUNCEMENT

Bacterial neonatal meningitis, one of the most devastating infections in the early period of human life, accounts for high mortality and morbidity among infants (1). *Escherichia coli* is the second most common pathogen associated with neonatal meningitis, and it accounts for 10% to 30% of these high mortality and morbidity rates, as well as adverse consequences in surviving neonates (1). Even though neonatal meningitis-causing *E. coli* (NMEC) has been considered one of the major pathogens associated with meningitis during the early period of human life, its pathogenesis has not been fully elucidated (2, 3). The NMEC strain RS218 (O18:H7:K1, ST95) was isolated from the cerebrospinal fluid of a patient with neonatal meningitis in 1974 (4, 5) . Over the past few decades, this strain of *E. coli* has been used extensively in the studies relevant to NMEC pathogenesis and is considered the prototypic strain of NMEC (3–5). Although some contigs of *E. coli* RS218 have been released, the complete and fully annotated genome of the RS218 strain is still not available (5). In the present study, the sequence of the genome of *E. coli* RS218, including its plasmid, was fully closed and completely annotated.

Genomic DNA of *E. coli* RS218 was isolated using the Promega Genomic Wizard kit (Madison, WI). Genome sequencing was performed with Ion Torrent PGM sequencing technology (Life Technologies, Grand Island, NY) at the Genomics Core Facility of The Pennsylvania State University (University Park, PA) using a 318 sequencing chip. The genome was assembled with both *de novo* and reference-guided assemblies using the DNASTAR SeqMan NGen v. 11.0.0 and Lasergene Suite (Madison, WI). The gaps were closed with primer walking, and the final assembly was anchored to an optical map generated by OpGen, Inc. (Gaithersburg, MD). Annotation was performed using both Rapid Annotation using Subsystem Technology and the NCBI Prokaryotic Annotation Pipeline (6, 7).

Analysis of the *E. coli* RS218 genome revealed that it consists of a circular chromosome of 5.087 Mb in size and a 114-kbp plasmid (pRS218) with an average G+C content of 50.6%. The sequence of pRS218 and its involvement in NMEC pathogenesis have recently been published (8). The chromosome contains 4,658 coding sequences, 88 transfer RNAs, 22 ribosomal RNAs, 1 clustered regularly interspaced short palindromic repeats array, and 5 noncoding RNAs. Additionally, it encodes secretory systems type I to VI (except the type III system), 8 fimbrial clusters, 6 iron acquisition systems, toxins, metabolic pathways, and several putative or hypothetical adhesins and invasins among other proteins. In comparison to the laboratory strain of *E. coli* K-12, the genome of RS218 contains 51 genomic islands which encode many known and potential virulence traits. These genomic data will be useful in future studies to broaden the current understanding of NMEC pathogenesis by identifying novel genes involved in initial colonization of mucosal epithelia as well as penetration of the intestinal mucosal barrier and blood-brain barrier by NMEC.

### Nucleotide sequence accession numbers.

The complete annotated chromosome and the plasmid of RS218 were deposited in NCBI GenBank under the accession numbers CP007149 and CP007150, respectively.
